# Distributions of easy axes and reversal processes in patterned MRAM arrays

**DOI:** 10.1038/s41598-023-47629-0

**Published:** 2023-11-22

**Authors:** William Frost, Robert Carpenter, Sebastien Couet, Kevin O’Grady, Gonzalo Vallejo Fernandez

**Affiliations:** 1 School of Physics, Engineering and Technology, University of York, Heslington, YO10 5DD UK; 2 imec, Kapeldreef 75, 3001 Leuven, Belgium

**Keywords:** Magnetic devices, Magnetic properties and materials, Spintronics

## Abstract

The distribution of the easy-axes in an array of MRAM cells is a vital parameter to understand the switching and characteristics of the devices. By measuring the coercivity as a function of applied-field angle, and remaining close to the perpendicular orientation, a classic Stoner-Wohlfarth approximation has been applied to the resulting variation to determine the standard deviation, $\sigma$, of a Gaussian distribution of the orientation of the easy-magnetisation directions. In this work we have compared MRAM arrays with nominal cells sizes of 20 nm and 60 nm and a range of free layer thicknesses. We have found that a smaller diameter cell will have a wider switching-field distribution with a standard deviation $\sigma = {9.5}^{\circ }$. The MRAM arrays consist of pillars produced by etching a multilayer thin film. This value of $\sigma$ is dominated by pillar uniformity and edge effects controlling the reversal, reinforcing the need for ever-improving etch processes. This is compared to larger pillars, with distributions as low as $\sigma = {5.5}^{\circ }$. Furthermore we found that the distribution broadens from $\sigma = {5.5}^{\circ }$ to $\sigma = {8.5}^{\circ }$ with free layer thickness in larger pillars and that thinner films had a more uniform easy-axis orientation. For the 20 nm pillars the non-uniform size distribution of the pillars, with a large and unknown error in the free-layer volume, was highlighted as it was found that the activation volume for the reversal of the free layer 930 nm$^3$ was larger than the nominal physical volume of the free layer. However for the 60 nm pillars, the activation volume was measured to be equal to one fifth of their physical volume. This implies that the smaller pillars effectively reverse as one entity while the larger pillars reverse via an incoherent mechanism of nucleation and propagation.

## Introduction

Magnetic Random-Access Memory (MRAM) is the most mature, non-volatile magnetic memory and is currently in production as an e-flash replacement^1,2^. The largest challenges to expand the application space for MRAM are: (1) the switching current, (2) data retention and (3) thermal stability^3^. All these are dependent on the anisotropy of the storage layer and its distribution. Any real-world magnetic system will consist of a combination of distributions and no individual parameter will have a uniform value. For example, the magnetisation will depend upon local composition and crystallographic effects or defects, or the nucleation field for reversal which will vary with dislocations, voids, inclusions and surface roughness. MRAM based on perpendicular magnetic tunnel junctions (p-MTJ) is no exception, especially for cell dimensions approaching 20 nm where there is a greater probability of pillar tilting, and device-to-device variation in the interfacial anisotropy. In this work we have focused on the distribution of the easy-axis of magnetisation perpendicular to the CoFeB in a p-MTJ.

The easy axis of CoFeB/MgO bilayers is often treated as a uniform vector, perpendicular to the plane. In reality, these vectors are distributed around a central maximum at ${90}^{\circ }$. According to the ubiquitous Stoner-Wohlfarth model for single domain entities, a small variation in the angle between the applied field, *H*$_{\mathrm a}$, and the anisotropy axis can significantly affect the coercivity of the film, *H*$_{\mathrm c}$. A deviation as small as ${10}^{\circ }$ from the easy axis of a prolate spheroid reduces the value of *H*$_{\mathrm c}$ by 30%^4^.

It is often assumed that the energy barrier to reversal, *E*$_{\mathrm B}$, is given by

1 $$\begin{aligned} E_B = KV \end{aligned}$$

where *K* is the anisotropy constant of the pillar and *V* its volume. However, due to the presence of the perpendicular demagnetising field, *H*$_{\mathrm D}$, this depends upon the ratio of *H*$_{\mathrm D}$ to the anisotropy field, *H*$_{\mathrm K}$, squared. *H*$_{\mathrm D}$ destabilises the magnetisation of the pillar and reduces the energy barrier to

2 $$\begin{aligned} E_B = KV \left[ 1 - \frac{H_D}{H_K}\right] ^2. \end{aligned}$$

As mentioned previously, all of these terms will be distributed themselves and can be imagined as $f_1(K)$ etc. $f_1(K)$ is a narrow distribution originating in small, local variations across the area of the pillar. These will be crystallographic due to dislocations, differing termination layers, diffusion or edge roughness. The distribution of volumes, $f_2(V)$, will similarly be narrow in a well defined MRAM array, with the small variation in pillar diameter *D* and thickness variations creating a distribution of *V*.

The distribution of *H*$_{\mathrm D}$, $f_3(H_D)$, is complex and has its origin in a number of sources. Any local edge damage, crystallographic defect or variation in aspect ratio will create a local variation of *H*$_{\mathrm D}$ on a pillar by pillar basis. As such this distribution is closely tied up with the others and will vary with them. We can mitigate some of these effects by measuring at the coercivity, where the magnetisation, *M*, is zero and therefore

3 $$\begin{aligned} H_D = -4\pi M = 0 \end{aligned}$$

across the whole film. However locally $M \ne 0$ and so the effects of *H*$_{\mathrm D}$ and $f_3(H_D)$ are unavoidable.

In this work we focused on the distribution in the anisotropy fields, $f_4(H_K)$, which originates from non-perfect alignment of the easy axes with the perpendicular direction. *H*$_{\mathrm K}$ itself as described by Luborsky^5^ is given by

4 $$\begin{aligned} H_K = \frac{\alpha K}{M_s} \end{aligned}$$

where $\alpha$ is a constant that describes the level of alignment of the easy axes with the applied perpendicular field in a magnetic volume. The value of $\alpha$ varies from 0.96 to 2.0 as the alignment improves from random to full alignment, hence the distribution of the easy axes is given by the distribution of $\alpha$, $f_5(\alpha )$. The misalignment of the easy axes will be twofold in origin: (1) the film itself will be granular and distributed and (2) the pillars will be non-uniform after etch. These anisotropies will be distributed in a Gaussian form around the ${90}^{\circ }$ direction with a defined standard deviation, $\sigma$. Using the Stoner-Wohlfarth model, previous work has calculated the switching field as a function of $\sigma$, in this instance for perpendicular media^6–10^. The effect of a broader distribution of *H*$_{\mathrm K}$ is to broaden the switching field distribution as a function of angle. The Stoner-Wohlfarth aligned case represents the narrowest possible distribution, i.e. an aligned case will be the most sensitive to the angle of an applied field *H*$_{\mathrm a}$. Any distribution in the value of *H*$_{\mathrm K}$ will therefore make the switching properties more variable from pillar to pillar in the MRAM final device. This also applies to the magnetic hardness mentioned previously. In order to have a robust, magnetically hard system the distribution of *H*$_{\mathrm K}$ must be as narrow as possible to minimise the effect of randomly oriented fields. While the destabilising effects of external AC fields can be compensated for by encapsulation, improving the longevity of the memory, long-term external DC fields applied to an MRAM array will have a strong angular dependence on the array and potential perturbation. As such measuring the distribution of *H*$_{\mathrm K}$ is also of importance with regards to the resistance of devices, magnetic noise and thermal activation.

It is important to note that additional distributions of pillar properties will arise when considering the electrical switching of MRAM pillars. Whilst the distributions mentioned are intrinsic to the reversal mechanism the electrical properties, such as the resistance-area product of a pillar *RA*, will have their own distributions e.g. *f*(*RA*) that go beyond the scope of this work but must be considered in a working device.

In this work we compared two different cell sizes and subsequently free-layer thicknesses, *t*$_{\text{FL}}$, for each diameter. The two pillar diameters were 20 nm and 60 nm and as such should exhibit very different switching field distributions. It is important to note the reversal mechanism controlling the reversal in these pillars. The most likely case is for an incoherent reversal of the spins within the pillar, similar to that discussed with regards to tape and longitudinal recording media^11^. The reality of the reversal is that a small volume will reverse at a localised defect due to a significant *H*$_{\mathrm D}$, followed by a rapid propagation of the domain wall through the pillar^12^. Such a reversal is called a nucleation controlled reversal and the switching field is a nucleation field, *H*$_{\mathrm N}$, as opposed to a more intrinsic coercivity. A brief introduction to the topic is given by Cullity^13^, but a more thorough treatment is given in the text by O’Grady et al.^14^.

The nucleation field is dominated by small features due to *H*$_{\mathrm D}$. This was exemplified in early particles used in the tape industry, where the switching field of an elongated ferrite particle ($\gamma$-Fe$_2$O$_3$) was limited to around 320 Oe . However, by applying a smoothing layer of cobalt ferrite (CoO$\cdot$Fe$_2$O$_3$) to the porous particle surface, the large local values of *H*$_{\mathrm D}$were reduced and the switching field increased by over 200% to 800 Oe^11,15^.

**Figure 1:**
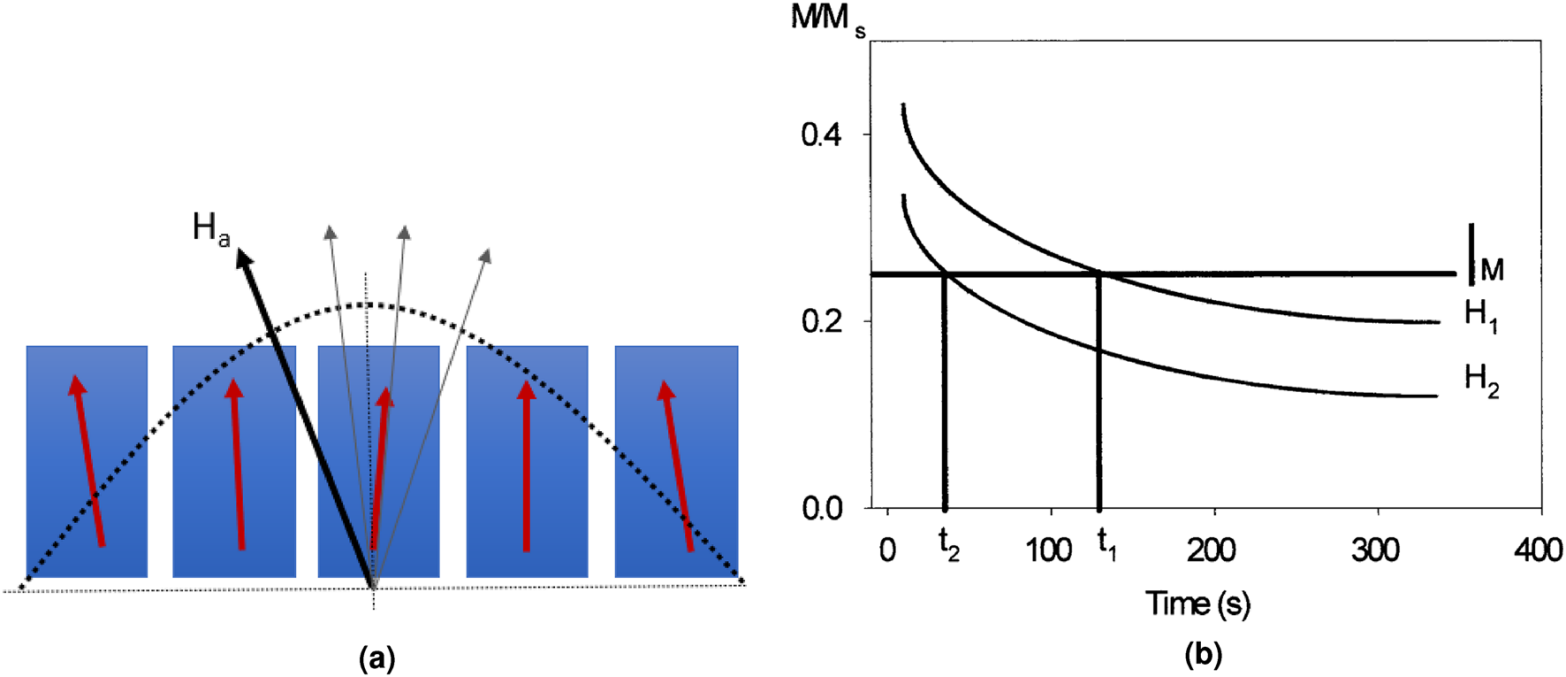
(**a**) The relative alignment of each pillar changes as *H*$_{\mathrm a}$ is swept across the normal direction, broadening the distribution of switching fields and lowering the reduction in *H*$_{\mathrm c}$ from the 30% predicted by the Stoner-Wohlfarth model. (**b**) Example data for a typical waiting time measurement of a continuous thin film, where the value of *t* is taken at a constant *M* under different values of *H*$_{\mathrm a}$, reproduced from^16,17^.

As in the work of Chureemart et al. on perpendicular disk media^6^, we have used the angular variation of values of the coercivity around ${90}^{\circ }$ to determine the switching field distribution. For well aligned particles such as these, a field range of $\pm {10}^{\circ }$ to the perpendicular is sufficient to give a large variation while having only a small effect on the demagnetising field. This allows for the Gaussian approximation to remain true and a simple model was used to fit the data given by

5 $$\begin{aligned} h = \frac{(1-t^2+t^4)^{\frac{1}{2}}}{(1 + t^2)} \end{aligned}$$

where $h = H_c(\theta )/H_c(0)$ and $t = tan(\theta )^\frac{1}{3}$^10^. The true 30% reduction predicted by Stoner-Wohlfarth is not observed because as some pillars come out of alignment, others come into it. As some pillars come out of alignment with the field, reducing their coercivity, others will come into alignment, increasing it. As such there will always be a balance of pillars aligned and misaligned with the applied field, broadening the distribution from the true Stoner-Wohlfarth case. This is shown in the schematic diagram in Fig. 1a.

A magnetic system is constantly experiencing thermal fluctuations, resulting in canting and oscillation of local spins. This can be imagined as an additional, destabilising field called the fluctuation field, *H*$_{\mathrm f}$, as proposed by Néel and improved upon by Street and Wooley^18,19^. This was introduced as a mechanism to couple the magnetic moment to thermal energy and was given by

6 $$\begin{aligned} H_f = \frac{k_BT}{Q} \end{aligned}$$

where $k_BT$ is the Boltzmann thermal energy and *Q* an undefined parameter. Later, Wohlfarth on dimensional grounds pointed out that *Q* must have dimensions of magnetic moment and rewrote Eq. (6) as

7 $$\begin{aligned} H_f = \frac{k_BT}{V_{act}M_s} \end{aligned}$$

where $V_act$ is an activation volume that nucleates the reversal and $M_s$ the saturation magnetisation^20^. This method only allows for the determination of $H_f$ when the time dependence is linear in *ln*(*t*). We have used the waiting time method described by el-Hilo et al.^16^ to determine *H*$_{\mathrm f}$ at a constant value of *M*, given by

8 $$\begin{aligned} H_f = \left. \frac{\partial H_a}{\partial ln(t)}\right| _M \end{aligned}$$

where *t* is the waiting time. This is the time *t* taken for the magnetisation of a film to reach a given value under a certain reversal field *H*$_{\mathrm a}$ around *H*$_{\mathrm c}$. A schematic diagram of the waiting time method is shown in Fig. 1b where a constant value of *M* intersects the time-dependence of *M* under two applied fields^17^. By using constant value of *M*, this allows for the effects of *H*$_{\mathrm D}$ to be ignored. The fluctuation field is then the rate at which the waiting time is influenced by the value of *H*$_{\mathrm a}$. This equation is modified from the equation of state of a magnetic material, developed by McCormick et al.^21^, at a constant value of *M*, for the rate of change of the field *H* at a changing magnetisation $\partial M/\partial t$ (=$\dot{M}$) given by

9 $$\begin{aligned} H_f = \left. \frac{\partial H}{\partial \dot{M}} \right| _M \end{aligned}$$

Using the value of *H*$_{\mathrm f}$ leads to an activation volume, *V*$_{\mathrm act}$, representing the size of the domain or grain that reverses in a single step under an applied field as defined by Wohlfarth^20^ in Eq. (7), before reversal occurs across the rest of the pillar given by

10 $$\begin{aligned} V_{act} = \frac{k_BT}{H_f M_s}. \end{aligned}$$

In the case of a nucleation controlled reversal, *V*$_{\mathrm act}$ is the volume of coupled spins that nucleate the reversal^22^. For a pillar with a smaller volume this can represent the free layer volume if it reverses coherently. For a larger pillar, nucleation at the edges reduces *V*$_{\mathrm act}$ as a fraction of the total free layer volume. The waiting time method is a time-dependence method, where the time taken for the magnetisation to reach a given value is taken under different starting conditions, i.e. different applied fields.

From previous work on these devices $M_s$ was taken as 1178 emu/cm^23^. These were then compared to the physical sizes of the pillars to discuss switching mechanisms in the films.

## Materials and equipment

The samples were deposited onto thermally oxidised 300mm Si-(001) substrates using a magnetron sputter deposition cluster tool (Canon-Anelva, EC7800). The samples were patterned into 60 and 20 nm diameter pillars with a centre-to-centre separation of 200 nm and 50 nm respectively. Printing of the masks for the pillar arrays was done using immersion for the 60 nm pillars and extreme-UV was used for the 20 nm pillars. The pattern was then transferred to produce the pillars using ion-beam etching, the process of which is described in previous work^24^. For magnetic characterisation, the arrays were diced into $\sim$4 × 4 mm squares along patterned scribe lines using a diamond saw of 50 μm width and placement accuracy of 5 μm, which was critical to ensure limited, or no unwanted magnetic material was present outside the array.

**Figure 2:**
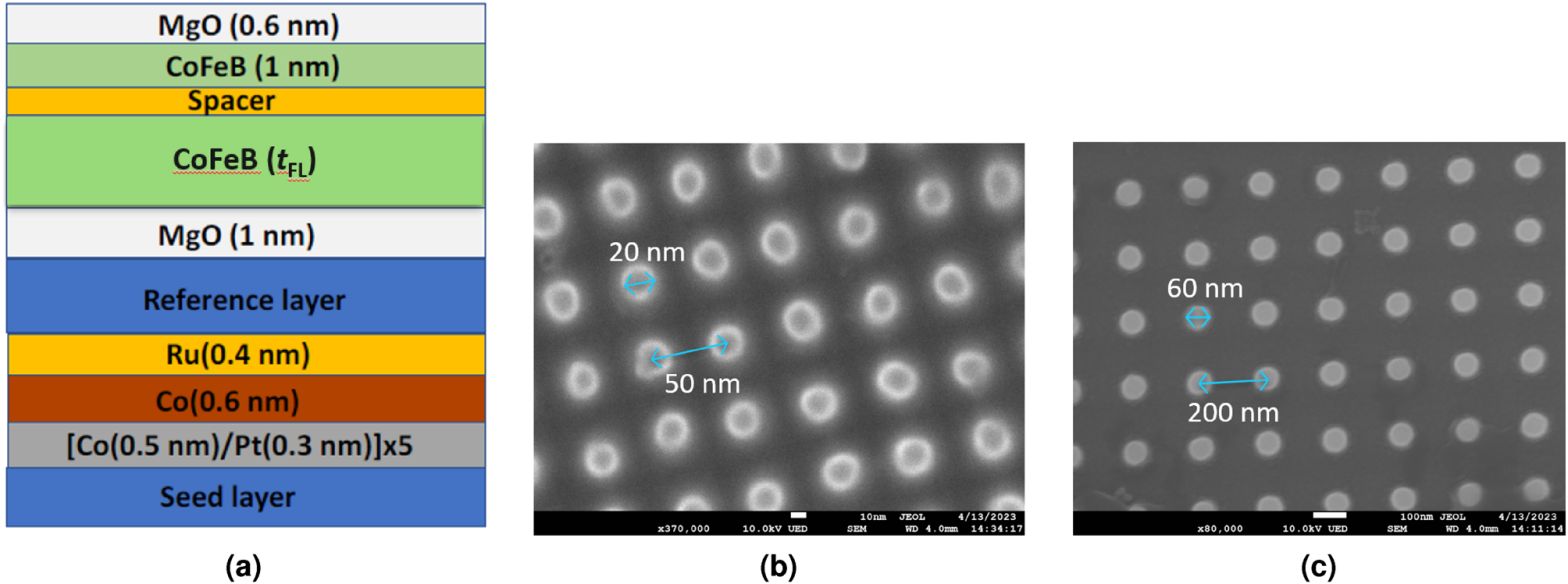
(**a**) The structure of the stack for patterning into MRAM pillars of (**b**) 20 nm and (**c**) 60 nm diameter pillars with centre to centre pitches of 50 and 200 nm respectively.

The structure of the samples was seed/[Co (0.5)/Pt (0.3)]$_{x5}$/Co (0.6)/Ru (0.4)/Reference Layer/MgO (1.0)/CoFeB (*t*$_{\text{FL}}$ $-1$)/Spacer/CoFeB (1.0)/Mg (0.6), where thicknesses are in nm . The reference layer is CoFeB and the spacer in the Free-Layer (FL) is a Ta oxygen scavenging layer to improve the crystallisation of the CoFeB and MgO, and therefore giving better magnetic properties of the pillars. The pillar structure is shown schematically in Fig. 2a with the spacer design which is described in a previous work^25^. In order to study the distribution of the switching fields, the effective anisotropy ($K_{eff}$) of the FL was varied by changing its thickness. This will serve to decrease the size of the interfacial anisotropy, $K_{i}$, which contributes to $K_{eff}$ as it scales according to 1/*t*^26,27^. Here, the total values of *t*$_{\text{FL}}$ chosen were 1.9 and 2.5 nm and 1.9 and 2.2 and 2.4 nm for the 20 nm and 60 nm pillars respectively. It is important to note that the reference and free layers will have their own sets of distributions such as *f*(V). In this work we are focusing on the free-layer distributions as these are the distributions explored in a device operation and it is to these that the schematic in Fig. 1a is referring.

The magnetic measurements were made using a LakeShore 8600 vibrating sample magnetometer (VSM) with a sensitivity of 1e−6 emu and a field precision of 0.1 Oe . The sample mounts allow rotation for measurement both in-plane and out-of-plane. The angular resolution of the rotation is ${0.1}^{\circ }$.

Micrographs were taken with a JEOL 8600F scanning electron microscope with a resolution of 1 nm, at an accelerating voltage of 10 KV and a working distance of 4 mm. Micrographs are shown for the two diameters of pillars in Fig. 2b and c with markings showing the nominal diameters of the pillars and the centre to centre pitches.

## Results and discussion

**Figure 3:**
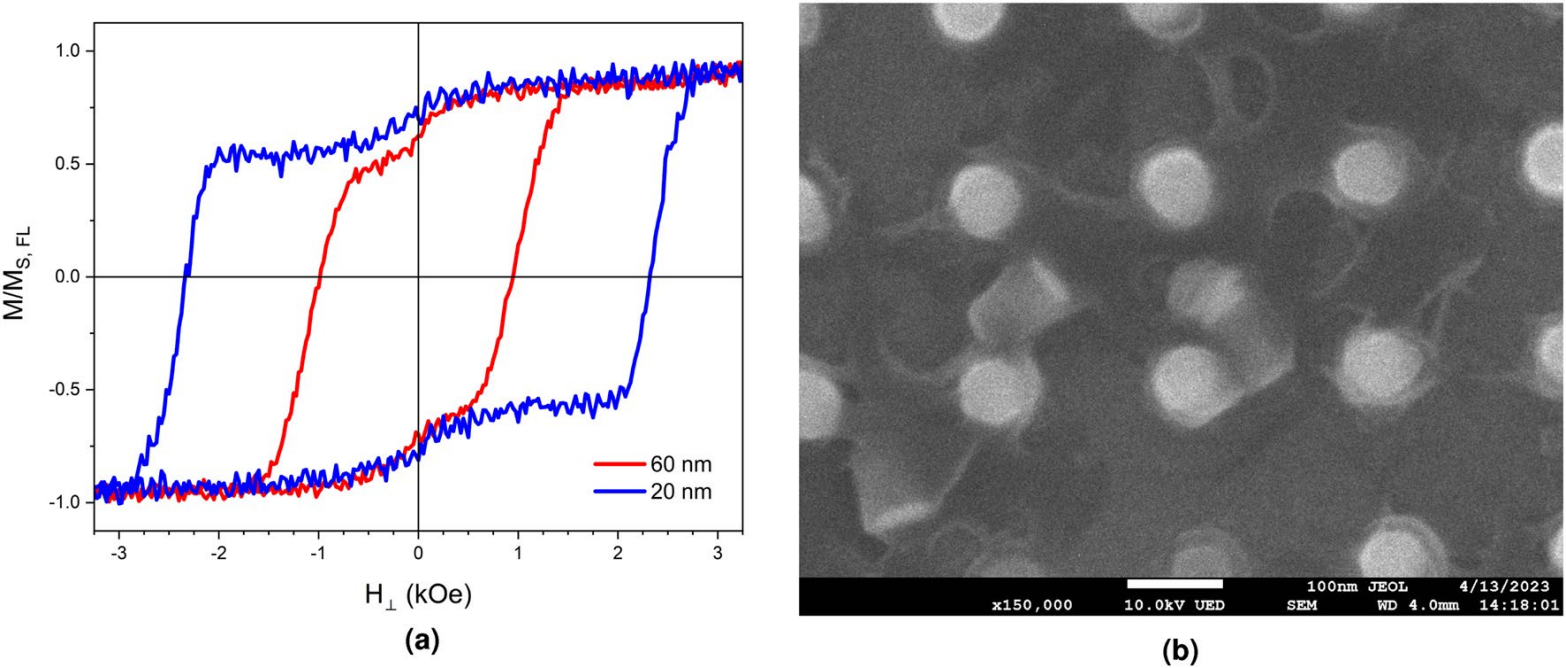
Out-of-plane *M-H* behaviour for the two different pillar diameters with a total *t*$_{\text{FL}}$ of 1.9 nm showing the rapid nucleation reversal.

Figure 3a shows the out-of-plane *M-H* loops for a typical array of pillars for each diameter with total free layer thicknesses *t*$_{\text{FL}}$ of 1.9 nm with *H*$_{\mathrm a}$ at ${90}^{\circ }$ to the plane of the sample. The switching region is very narrow, indicating the rapid nucleation controlled reversal discussed above. The low-coercivity region in the centre of the loop is due to the some of the pillars toppling due to the vibration in the magnetometer or an electrostatic discharge toppling the pillars as shown in Fig. 3b. The pillars in this work are not encapsulated in SiO$_2$ or SiN and hence are free standing. The toppled pillars have an in-plane anisotropy and therefore little or no coercivity out-of-the-plane so do not contribute to the switching field distribution in the perpendicular orientation. Additionally, as the pillars are not encapsulated there is a random oxidation layer on the surface of the pillar. The formation of oxides will affect the local demagnetising fields stochastically on each pillar and may introduce new anisotropy through pinning or an exchange bias, if CoO is present in an antiferromagnetic phase. This will act to further broaden $f_4(H_K)$, especially in the smaller area, 20 nm pillar array.

It is important to note that this data is presented with the applied field not corrected for *H*$_{\mathrm D}$. This is because of coupling effects, whereby a correction of $4\pi M$ results in an over-correction and a shearing of the loop. However, the data is representative of the true loop of the pillars in use and is that which controls the thermal behaviour. The narrow width of the switching region between 2.25 and 2.75 kOe indicated a narrow switching field distribution, and therefore the $\pm {10}^\circ$ variation in the applied field should be sufficient. There is no evidence in the hysteresis loop of direct coupling between the layers in an individual pillar. However, dipole-dipole interactions are inevitably present by cannot be quantified and thus how this affects the activation volume of each pillar is also not quantifiable. The asymmetries in the loops are also caused by the additional layers introducing slight offsets, possibly related to the dipole-dipole interactions.

**Figure 4:**
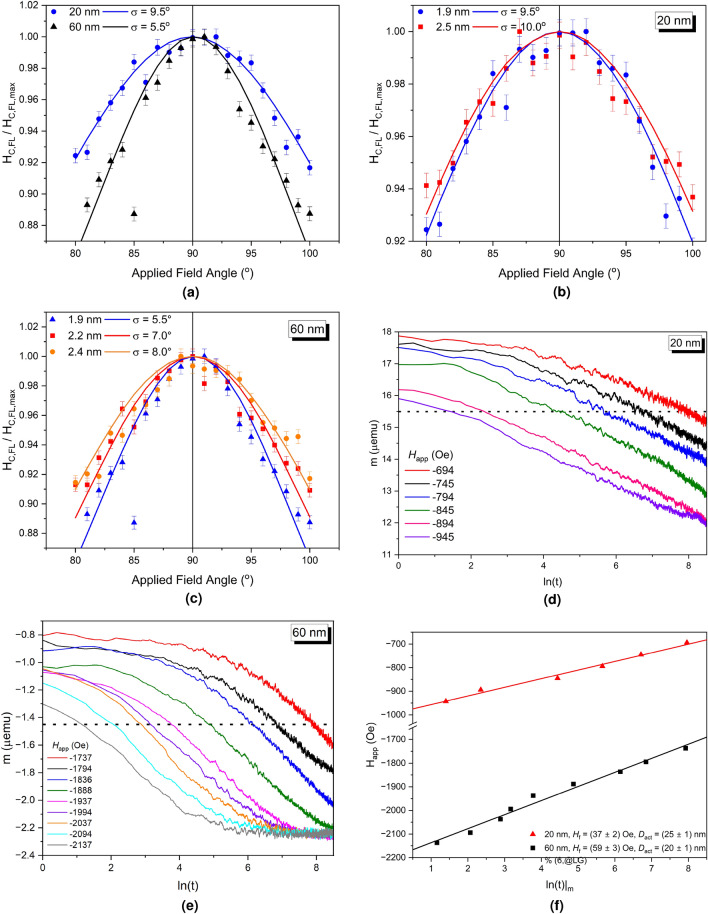
(**a**) The angular variation in the coercivity for 20 and 6 nm pillars with a *t*$_{\text{FL}}$ of 1.9 nm with a fit to Eq. (5). (**b**) The easy axis distributions as function of applied field angle for the 20 nm pillars and (**c**) 60 nm with varying *t*$_{\text{FL}}$. (**d**) Time dependence measurements at varying applied fields for the (**d**) 20 nm and (**e**) 60 nm pillars with *t*$_{\text{FL}}$ = 1.9 nm . (**f**) The dependence of *H*$_{\mathrm a}$ on ln(t) to reach a specific magnetic moment, the gradient of which gives *H*$_{\mathrm f}$, where *m* = − 14.5$\mu$ emu and *m* = − 1.45$\mu$ emu emu for the two samples respectively denoted by the dotted line.

Figure 4a shows the variation in the switching field at $m = 0$ as a function of the angle of *H*$_{\mathrm a}$ for the samples having two pillar diameters and a thickness of the free layer *t*$_{\text{FL}}$ = 1.9 nm . The curves are normalised to the maximum value of the coercivity of the free layer, $H_{C, FL}$, for each individual array of pillars for ease of comparison. It is immediately obvious that the smaller, 20 nm pillars have a wider distribution, with $\sigma = {9.5}^\circ$ as opposed to $\sigma = {5.5}^{\circ }$ for the larger pillars. This possibly arises as *H*$_{\mathrm N}$ for a smaller pillar is dominated by the edge roughness. Any defect will represent a significant proportion of the volume of the pillar and as such will be the driving factor for reversal. There is an additional source of variation in the local value of *H*$_{\mathrm K}$ from the angular variations in smaller pillars. The long axis of the pillars will be more distributed around the perpendicular, but it is probable that this is overshadowed by the variations in edge roughness and edge domain formation. Additionally the variation in *f*(*V*) will be more significant in smaller pillars due to a much broader size distribution post-etch and these variations are clearly shown in Fig. 2b as compared to Fig. 2c. For larger pillars these two effects are reduced so there is behaviour closer to the Stoner-Wohlfarth case. As such the local Stoner-Wohlfarth process on each pillar is much more susceptible to a variation in *H*$_{\mathrm K}$, as described in Fig. 1a.

Figure 4b and c show the dependence of the easy axis distribution on *t*$_{\text{FL}}$. For the 20 nm pillars the change is small with $\sigma$ remaining largely unchanged. This confirms the effect being dominated by the edge roughness effects and the distribution is so dominated by these that it is mostly independent of other parameters. A much stronger dependence on *t*$_{\text{FL}}$ is observed for the larger pillars, however, where an increase in *t*$_{\text{FL}}$ sees a broadening of the distribution, from $\sigma = {5.5}^{\circ }$ to $\sigma = {8.0}^{\circ }$ when *t*$_{\text{FL}}$ reaches 2.4 nm. The reason for the pillars increasing in angular stability with thickness is unclear. However, since the total volume of the pillar is increasing so is the total moment. Any cross-talk will be increased by this increase in moment, which may act to stabilise neighbouring grains and hence broaden the distribution.

Figure 4d and e show the time dependence data for the two different diameters with *t*$$_{\text{FL}}$$ of 1.9 nm . The intercept point at a constant value of *m* is taken in *ln*(*t*) for each *H*$$_{\mathrm a}$$  where $$m = $$ − 14.5$$\mu $$ emu and $$m = $$ − 1.45$$\mu $$ emu for the two samples respectively, denoted by dotted lines. Note that due to the presence of the reference layer, *m* never reaches zero around the value of *H*$$_{\mathrm c}$$ of the free layer, meaning that *H*$$_{\mathrm D}$$ is non-zero. However, all measurements are taken at constant *m* and therefore a constant *H*$$_{\mathrm D}$$  so the effect at each value of *H*$$_{\mathrm a}$$ is the same and can be ignored. Due to the presence of the reference layer and local variations in local interactions and properties, the values of *H*$$_{\mathrm K}$$ and *H*$$_{\mathrm D}$$ cannot be known for an individual free layer or pillar. The time dependence data are then plotted for each field in Fig. 4f and the gradient taken, giving the value of *H*$$_{\mathrm f}$$ from Eq. (8). For the pillars with a diameter of 20 nm the value of *H*$$_{\mathrm f}$$ was 37 ± 2 nm which increases to 59 ± 3 nm for the larger pillars. It is important to note the difference in the non-linearity in the two figures Fig. 4d and e. The non-linearity of the larger pillars is more pronounced, indicating a narrower switching field distribution. Only for a broad distribution does linear ln*(t)* behaviour occur, whereas narrow distributions result in non-linear behaviour^16^.

For the case where *M* varies linearly with *ln*(*t*)

11 $$\begin{aligned} M(t) = M(0) \pm S(H)ln(t) \end{aligned}$$

*S*(*H*) is the time dependence coefficient^19^. This coefficient is then described by^28^

12 $$\begin{aligned} S(H) = \left. 2M_skTf(\Delta E)\right| _{\Delta E_c(H)} \end{aligned}$$.

**Figure 5:**
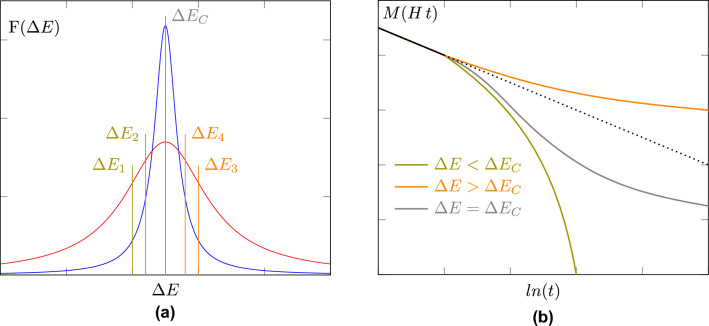
(**a**) A schematic of a narrow and broad distribution of energy barriers where $\Delta E$ represents which particles are reversing as time progresses. (**b**) A schematic showing how the time dependence on either side of the coercivity deviates from non-linear ln(t) behaviour because of the narrow distribution having a rapid change in F($\Delta E$).

This is shown schematically in Fig. 5a, where the time dependence of energy barrier reversal is shown for two different distributions $f(\Delta E)$ of the energy barriers, $\Delta E$. At *H*$_{\mathrm a}$< *H*$_{\mathrm c}$, the grains being reversed have an energy barrier $\Delta E_1$. As time passes through the waiting time experiment, the grains being reversed progress to those at $\Delta E_2$ and so forth. All of the values in Eq. (12) can held constant and the time-dependence is affected only by the value of the distribution function at the critical energy barrier at a given field. For a wide distribution, shown in red, this results in a small increase in the number of grains being reversed as the values of $df(\Delta E)/d(\Delta E)$ are small. The result is quasi-linear behaviour in ln*(t)*. However, a rapid increase in $f$ $(\Delta E$) is observed in a narrow distribution shown in blue, where $df(\Delta E)/d(\Delta E)$ is large. As such the rate of change in magnetisation increases rapidly as the value of *f*($\Delta E$) approaches its maximum at $\Delta E_C$. This is shown in Fig. 5b by the green line, where $\partial M/\partial ln(t)$ rapidly increases. The opposite is true above *H*$_{\mathrm c}$, as shown by $\Delta E_{3,4}$ and the orange line. The decreasing volume fraction of the sample means that the change in magnetisation decreases over time, as fewer and fewer grains are reversed. At values approaching *H*$_{\mathrm c}$, a combination of the two mechanisms is seen, shown in grey in Fig. 5b^16^.

Figure 4d shows, therefore, that the smaller pillars have a broad switching field distribution as expected from the data in Fig. 4b. The slight curvature is consistent with the measurements being slightly below *H*$_{\mathrm c}$, and the non-linearity decreases with *H*$_{\mathrm a}$ as expected. Figure 4e shows a very narrow switching field distribution is present in the larger pillars, as the significant increase in *dm*/*dt* shows. As *H*$_{\mathrm a}$ approaches *H*$_{\mathrm c}$, the behaviour switches towards that shown in grey in Fig. 5b, as expected.

Figure 4f compares the value of the time-taken to reach a specific magnetisation given a different *H*$_{\mathrm a}$ approaching *H*$_{\mathrm c}$. The gradient of this gives the fluctuation field, *H*$_{\mathrm f}$, and from this an activation volume can be calculated and the results are summarised in Table 1. For the smaller pillars the value of *V*$_{\mathrm act}$ is larger than their nominal physical volume. This highlights the wide distribution of pillar sizes and shapes present in the arrays. The results are consistent with a form a quasi-coherent reversal taking place as the entire pillar behaves as one magnetic entity. For the larger pillars the value of *V*$_{\mathrm act}$ is smaller, and significantly smaller than the pillar physical volume. This represents a nucleated domain at the edge of the pillar, where a 600 $\text{nm}^{3}$ volume represents 21% of the total volume of a 60 nm pillar ($\sim$ 2800 $\text{nm}^{3}$). This small edge domain then propagates rapidly through the pillar, as shown in Fig. 3 and must be due to incoherent reversal. The origins and physics of the exact nature of this reversal are unclear. The higher angular sensitivity could be due to the distributed nature of these edge regions of activation. Each region will have its own anisotropy and the misalignment of any one of these will result in a nucleated reversal at a reduced field. Therefore a small change in the applied field angle is more statistically likely to result in an activation in a larger pillar.

**Table 1:** The values of *H*$_{\mathrm f}$ for each value of the pillar diameter with a value of *t*$_{\text{FL}}$ = 1.9 nm and subsequently the calculated values of *V*$_{\mathrm act}$.

Pillar diameter (nm)	*t*$_{\text{FL}}$ (nm)	Free-layer volume ($\text{nm}^{3}$)	*H*$_{\mathrm f}$ (Oe)	*V*$_{\mathrm act}$ ($\text{nm}^{3}$)	*D*$_{\mathrm act}$ (${\text{nm}}^{2}$)
20	1.9	600	$37 \pm \ 2$	$930 \pm \ 40$	$25 \pm \ 2$
60	1.9	2830	$59 \pm \ 3$	$600 \pm \ 20$	$20 \pm \ 1$

The total volume of the free-layer and the diameter of the activation volume, *D*$_{\mathrm act}$, are shown for ease of comparison to the values of *V*$_{\mathrm act}$ and the pillar size.

## Conclusions

We have shown that the angular variation in the switching fields of pillars is broadened compared to the classical Stoner-Wohlfarth case in 20 nm and 60 nm pillars. Smaller pillars have a broad switching field distribution, dominated by edge damage and etch-induced variations as the surface of the pillar represents a significant volume of the pillar and the etch process is poorer overall. Larger pillars are closer to a classical case, but still broadened by a $\sigma$ of ${5.5}^{\circ }$ to ${8.0}^{\circ }$ depending on the thickness of the free-layer. The narrower switching field is demonstrated through a significant non-linearity in the time dependence data. From time-dependence data we have shown that the activation volume of the pillars is similar despite the large difference in the volumes of the pillars. This confirms the nucleation controlled reversal suggested by the hysteresis loops and that a macro-spin approximation is not valid in larger pillars. The nucleation and reversal in the pillars is dominated by the surface roughness. Furthermore the evidence of nucleation and propagation controlled reversal requires a reexamination of the macro-spin approximation for smaller pillars with D $\approx$20 nm. The exact boundary where macrospin failure occurs needs further investigation across a wide range of pillar diameters. This has significant implications for the modelling of future MRAM architectures and for investigating the magnetic hardness and reliability of MRAM.

## Data Availability

The datasets used and/or analysed during the current study available from the corresponding author on reasonable request
